# Devising a deep neural network based mammography phantom image filtering algorithm using images obtained under mAs and kVp control

**DOI:** 10.1038/s41598-023-30780-z

**Published:** 2023-03-02

**Authors:** Sung Soo Park, Young Mi Ku, Kyung Jin Seo, In Yong Whang, Yun Sup Hwang, Min Ji Kim, Na Young Jung

**Affiliations:** 1 Deargen Inc., Daejeon, 34051 Republic of Korea; 2 Department of Radiology, Uijeongbu St. Mary’s Hospital, College of Medicine, The Catholic University of Korea, Seoul, Republic of Korea; 3 Department of Hospital Pathology, Uijeongbu St. Mary’s Hospital, College of Medicine, The Catholic University of Korea, Seoul, Republic of Korea; 4 Department of Radiology, Uijeongbu Eulji Medical Center, College of Medicine, Eulji University, Uijeongbu, Gyeonggi-do Republic of Korea

**Keywords:** Computational biology and bioinformatics, Health care, Medical research

## Abstract

We study whether deep neural network based algorithm can filter out mammography phantom images that will pass or fail. With 543 phantom images generated from a mammography unit, we created VGG16 based phantom shape scoring models (multi-and binary-class classifiers). Using these models we designed filtering algorithms that can filter failed or passed phantom images. 61 phantom images obtained from two different medical institutions were used for external validation. The performances of the scoring models show an F1-score of 0.69 (95% confidence interval (CI) 0.65, 0.72) for multi-class classifiers and an F1-score of 0.93 (95% CI 0.92, 0.95) and area under the receiver operating characteristic curve of 0.97 (95% CI 0.96, 0.98) for binary-class classifiers. A total of 42 of the 61 phantom images (69%) were filtered by the filtering algorithms without further need for assessment from a human observer. This study demonstrated the potential to reduce the human workload from mammographic phantom interpretation using the deep neural network based algorithm.

## Introduction

Screening out definite passed or failed mammography phantom images ahead of visual scoring would reduce the radiologist’s workload for quality assessment. The purpose of this study was to develop a Deep Neural Network (DNN) based filtering algorithm for screening mammography phantom images and to evaluate its feasibility.

In Korea, the phantom image evaluation should be performed every 6 months; it has been included in the annual and 3-year cycle inspection by accrediting agencies. Considering that, as of 2016, there are 3138 mammography units in operation in Korea, approximately 6000 evaluations are being conducted annually by accrediting agencies. Furthermore, most hospitals are actually managing phantom mammography more frequently according to their manufacturers’ quality control manual, so that countless tests are being conducted every day nationwide. The Mammography Quality Standards Act requires weekly tests as the minimum frequency, hence more tests are expected if quality control improves to the level of that in the United States. In short, the numerous phantom mammography performed on a regular basis place a workload on the radiology personnel, and these examinations will increase in the future. Therefore, the authors designed a filtering algorithm using a DNN to interpret the phantom images, with the aim of reducing the workload of the radiology personnel.

Many attempts have been made to perform automated analysis of phantom images^1–5^. Generally, these studies consist of two steps. First, the image preprocessing step is a process of localization of target wax inserts and enhancing target visibility using mathematical formulas and digital image processing techniques such as Fourier-domain template matching, discrete wavelet transform, and Sobel filter. Next, the image evaluation step is a process in which measurement and classification of localized objects are performed. Bayesian classifier, Mahalanobis distance, and cross-correlation methods were used. By demonstrating reliable agreement between computerized scoring and human scoring, the authors proved automated phantom scoring is also possible. Previous studies just calculated the quantitative values of designated key features (length, number, roundness, and so on) related to the phantom shape scoring through mathematical methods. However, a phantom image is a sort of a complex radiographic image composed of various densities and contrasts. Thus, defining only selected features is not sufficient to interpret a phantom image. The current method of a DNN greatly differs from such conventional analysis. Extracting higher-level features necessary for scoring would happen if trained through a DNN. Thus, a more sophisticated phantom interpretation would be possible in the end. This is the background of the present machine learning-based approach.

Recently, there have been two studies of American College of Radiology (ACR) phantom image quality assurance using DNN. Oh et al.^6^ developed and validated an interpretable deep learning model using a large and diverse dataset to objectively evaluate the quality of standard phantom image. Recognition of fiber, speck, and mass evaluation models showed high performance and reasonable object scoring. Therefore, the proposed model can serve as an assistant tool for evaluating mammography phantom images. Ho et al.^7^ designed an image quality evaluation architecture that accurately predicts human evaluations. The minimum training sample size for the model’s complexity and the most influential feature were also determined. However, no attempt was made to reduce the reader's workload. Unlike previous studies that attempted to assist the visual scoring, our aim was to reduce the overall workload by eliminating passed or failed films using a specific algorithm (Table 1). Our attempt is something new that no one has ever done before. Our study will show the possibility that phantom studies using DNNs are not limited to implementing scoring accuracy but can be extended to other clinical applications.

**Table 1:** Comparison with the previous ACR phantom studies.

	Oh et al.^6^	Ho et al.^7^	Current study
Purpose	Objective evaluation of phantom images	Objective evaluation of phantom images	Reducing workload by filtering phantom images
Dataset	2,208 images (from 1,755 institutions)	461 images (10 mammographic system, 1 ACR phantom)	640 images (1 mammographic system, 1 ACR phantom)

Model	YOLO v2	SVM	VGG16
Accuracy	fiber:specks:mass = 0.995:0.993:0.998 overall, 0.995	fiber:specks:mass = 0.902:0.982:0.889 overall, 0.924	fiber:specks:mass = 0.877:0.951:0.751 overall, 0.861 (multi-class classifier) fiber:specks:mass = 0.893:0.951:0.908 overall, 0.916 (binary-class classifier)
Note	Large and diverse dataset	Minimum sample size experiment for model complexity (30% of the total data set) Most influential feature (position feature)	Phantom image filtering trial with threshold controlled algorithm (69% of filtering rate)

ACR, American College of Radiology; YOLO v2, You Only Look Once version 2; SVM, Support Vector Machine; VGG16, Visual Geometry Group 16.

## Materials and methods

**Figure 1:**
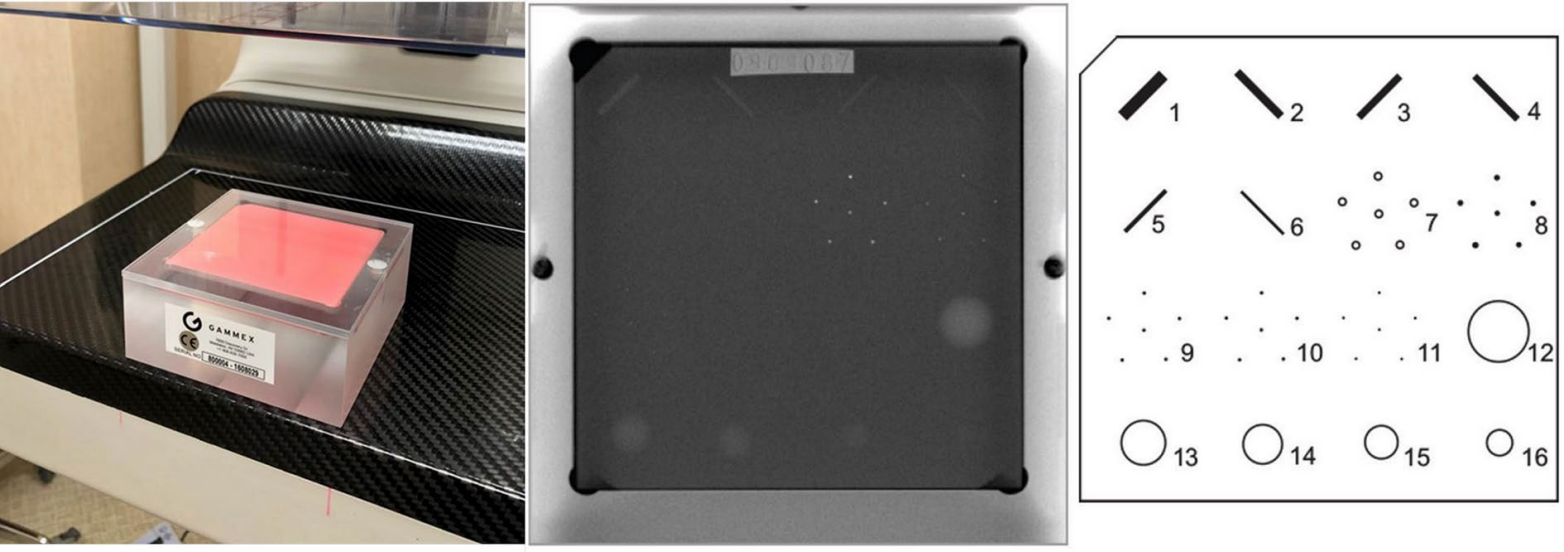
Mammographic accreditation phantom, phantom image, and diagram. American College of Radiology (ACR) phantom (1999 version) containing simulated lesions of 6 fibers, 5 speck groups, and 5 masses.

Mammographic phantoms are used to monitor the ability of the mammography unit to image small detailed structures similar to those found clinically and ultimately to assure that the image acquisition chain is consistently producing adequate image quality at optimum levels. Changes in the phantom image is a signal that one or more of the components in the imaging chain are below the quality standards. The source of the problem should be identified and corrective action needs to be taken before any further examinations are performed (Fig. 1).

This study does not involve human subjects. Our research was not subjected to deliberation by the participating institutional review boards.

### Study concept and outline

**Figure 2:**
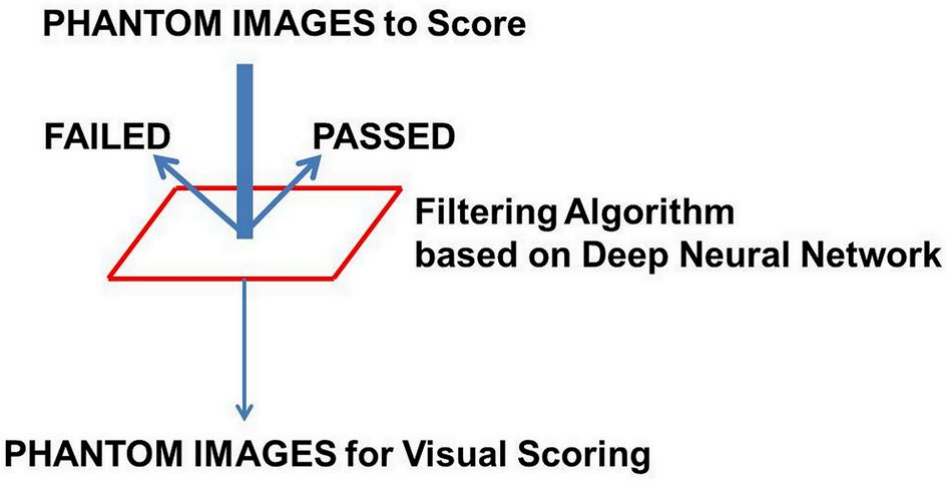
Study concept for reducing the phantom scoring workload. The thickness of the arrow indicates the volume of the image to interpret.

If there was a DNN-based algorithm that could filter out passed or failed phantom images in advance, the number of images for direct visual scoring could be reduced (Fig. 2). Therefore, based on a DNN, we created a model that allows for scoring of each shape shown in the phantom image. We then designed an algorithm that can filter a passed or failed phantom image based on that model. Finally, by applying the proposed filtering algorithm to phantom images obtained in quality control, we examined whether the number of images for visual scoring could actually be decreased.

### Phantom shape scoring models

#### Data collection

Phantom images, generated from a mammography unit (Selenia® Dimensions® Mammography System, Hologic, USA) and a mammographic accreditation phantom (Mammo 156 ™ Phantom, Gammex, USA) used in our institution, are usually taken at approximately 130–150 mAs and 28 kVp. Phantom images are more formalized than clinical radiography. However, in order to obtain as wide a variety of phantom images as possible from the current equipment, we have used a full range of mAs (60–220)/kVp (22–32) capable of image generation. By step-wise adjustment of radiation exposure factors (mAs, kVp), phantom images were obtained within all mAs (60–220) and kVp (22–32) intervals. In our mammography unit, we can manually adjust the mAs in two-unit intervals and the kVp in one-unit intervals. Theoretically, we can obtain 880 images [(16 × 5) mAs × 11 kVp]. However, similar units would then produce nearly identical images. Therefore, except where possible, only 2–4 images (not every 5 images) were obtained from the combinations of mAs and kVp in each section. The mammography phantom is made of a rigid, non-compressible 4.5 cm thick acrylic material (4.5 × 10.2 × 10.8 cm). Therefore, the compression pressure is always constant and the thickness cannot be changed. In our equipment, a compression paddle with a size of 18 × 24 cm is always used for phantom imaging, and the field of view is always constant at 18 × 24 cm (2560 × 3328 pixel). In phantom imaging, auto-filter mode (rhodium or silver) is basically applied, but rhodium filter is always adopted due to the constant phantom thickness (4.5 cm). The target of the X-ray tube is only the tungsten. For mammographic accreditation phantom imaging, the automatic exposure control sensor (AEC) is placed under the phantom model. The accreditation phantom is also constant, but the kVp and mAs regulated by the AEC vary slightly each time it is shot. Therefore, phantom images were obtained by varying only two factors. We have excluded the phantom images which were too white or black due to a combination of too low or high exposure factors. A total of 640 phantom images remained for use in our study (Supplementary Table S1). As we intended to generate as many images as possible for the training, the phantom images were generated regardless of the adequacy of the background optical density.

One radiologist was able to review 640 phantom images, where some that were not deemed suitable for scoring were excluded, such as those with the phantom shape (fiber, speck, and mass) being severely distorted or being in an incorrect position. Finally, 543 phantom images [3,258 (543 × 6) fibers, 2715 (543 × 5) specks, and 2715 masses] were included in the development set of our study.

**Figure 3:**
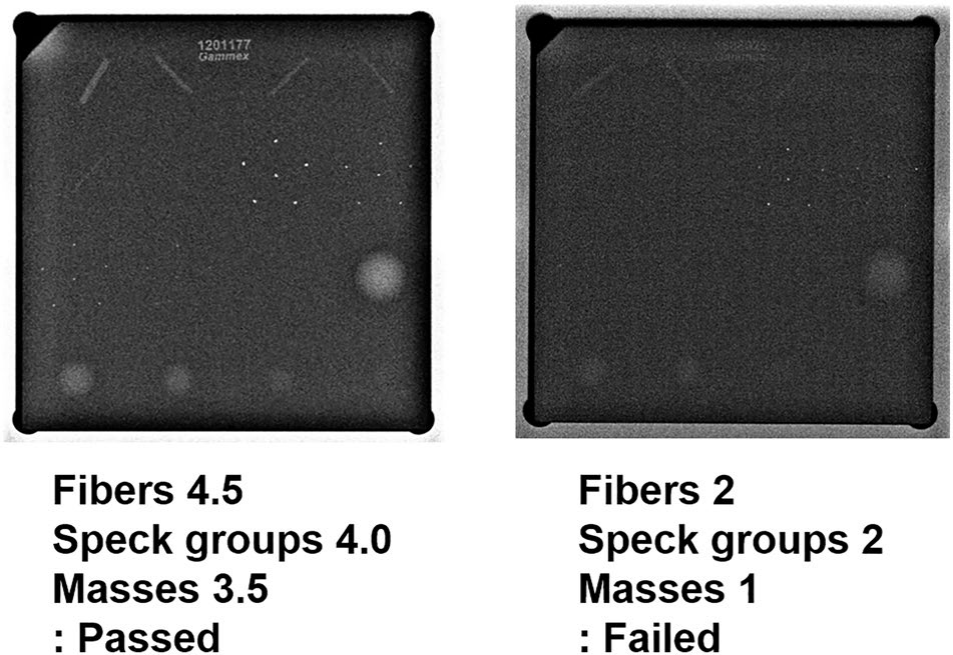
Examples of American College of Radiology (ACR) phantom scoring. At a minimum, the four largest fibers, the three largest speck groups, and the three largest masses must be visible.

Each phantom image was displayed on a 5-megapixel, dedicated mammography monitor and was adjusted to the most readable window/width level based on a consensus of two radiologists (among IY Whang, YS Hwang, MJ Kim and NY Jung). Each phantom shape from the 543 phantom images was displayed on a monitor and was scored simultaneously by the two radiologists according to the ACR phantom scoring method^8^. Brief scoring criteria and actual phantom scoring examples are provided (Supplementary Table S2, Fig. 3). If the scores differed, a consensus score was assigned to one shape. Scoring all 16 shapes in a single phantom image took less than 1 min, but we did not score more than 25 phantom images per session.

#### Training of the models

When a phantom image was received, we set a reference point, and the shape of a 224 × 224 pixel was automatically cropped and extracted at the region of interest where the shape was located. Then, the intensity of the image was rescaled to improve the contrast. The partitioning of development data was performed for each shape (3258 fibers, 2715 specks, and 2715 masses). All of the extracted shapes were then mixed and randomly assigned into three data sets, which will be used for training, validation, and holdout. A component used in one data set was not used in another data set, for example, one fiber is used once for one of each data set. The development data set was divided into training/holdout (80:20), and the training data was further divided into training/validation (80:20). As a result, the ratio of training/validation/holdout sets was 2084:522:652 (fibers) and 1,737:435:543 (specks, masses), showing 64%:16%:20% (Table 2).

**Table 2:** Development data set status and score.

543 Phantom images				
	No			
	Score	Fibers	Specks	Masses
Development set		3258	2715	2715
Training		2084	1737	1737
	0	528 (25%)	430 (25%)	541 (31%)
	0.5	156 (3%)	60 (3%)	472 (27%)
	1	1400 (67%)	1247 (72%)	724 (42%)
Validation		522	435	435
	0	135 (26%)	99 (23%)	130 (30%)
	0.5	36 (7%)	13 (3%)	119 (27%)
	1	351 (67%)	323 (74%)	186 (43%)
Holdout		652	543	543
	0	176 (27%)	126 (23%)	167 (31%)
	0.5	50 (8%)	23 (4%)	154 (28%)
	1	426 (65%)	394 (73%)	222 (41%)

**Figure 4:**
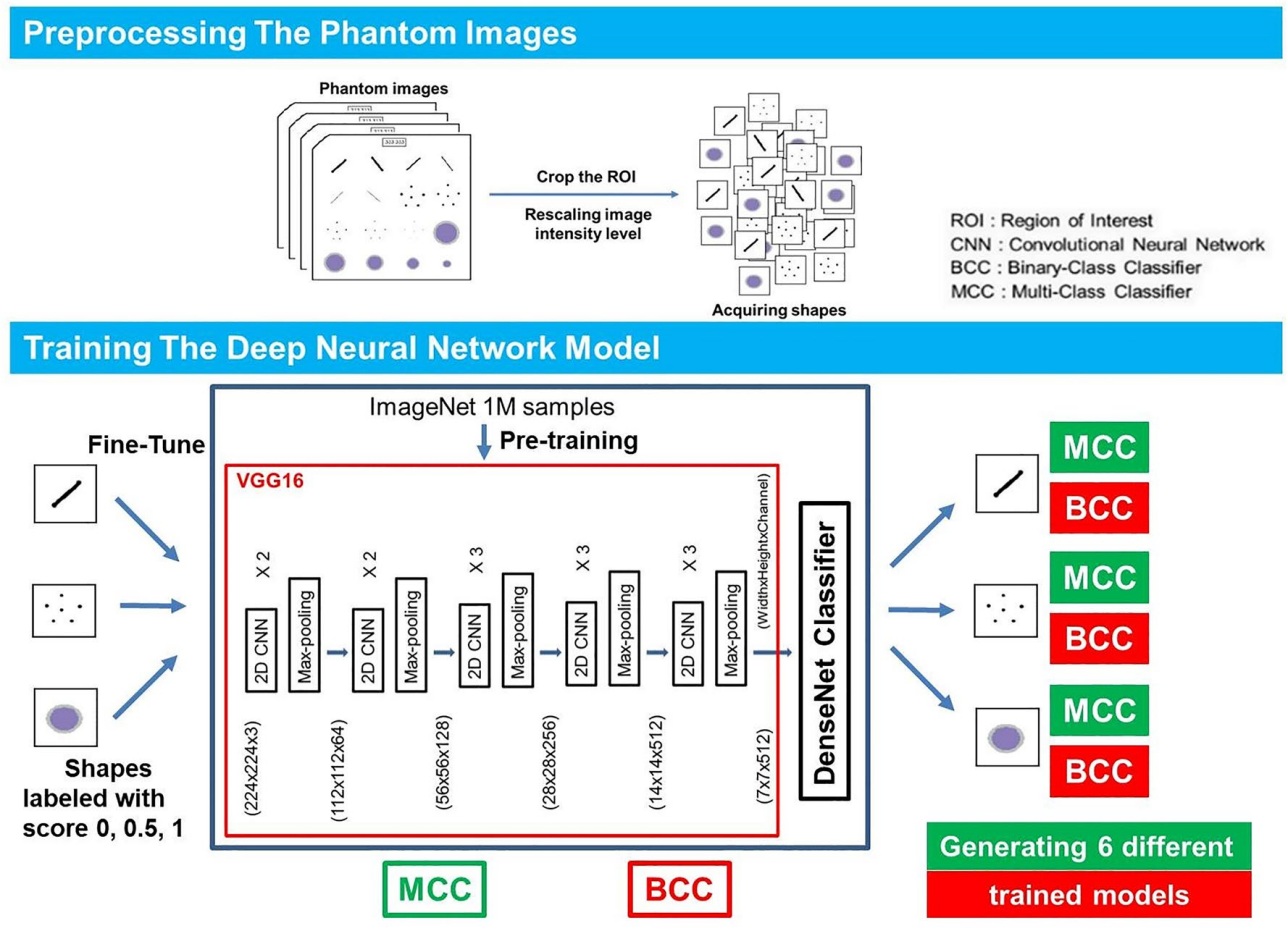
The whole process and the architecture of the deep neural network based phantom shape scoring models. In the preprocessing, only the part of the shape to be scored is cut out. Then, adjust the intensity to take advantage of the features of the shape. A deep neural network model was then modified using a VGG16 model based on the 2D Convolutional Neural Network. After loading the model weights learned in ImageNet 1 million images, the phantom shape image is fine-tuned. Binary-class classifiers (BCC) and multi-class classifiers (MCC) models are finally learned for each shape according to the outcome labeling. The BCC model predicts whether the shape will or will not be scored. The MCC model predicts a score of 0 or 0.5 or 1 for the shape.

Before designing the DNN model, performance comparisons between several models (VGG16 vs. ResNet50 vs. MobileNet) were conducted. VGG16 has a total of 138 million trainable parameters (vs. ResNet50, 23 million vs. MobileNet, 13 million). The complexity of VGG16 is higher, but it was used for its accuracy (Supplementary Table S3). A DNN model was constructed using the basic model of VGG16. VGG16 was used as it is in the feature extraction part of the model architecture, and multi (0 or 0.5 or 1)- and binary (0 or not)-class classifications were applied to the output layer to be suitable for phantom scoring. For one shape, two models with different output layers were constructed respectively. The pre-training of a DNN model on more than one million images from ImageNet was conducted. Pretraining was slightly better than training from scratch (reset weight) (Supplementary Table S4). The generalizability was improved by using weights (transfer learning) that were previously learned in about 1 million image classification tasks and the number of samples was increased by performing data augmentation (horizontal and vertical flip). A dropout layer was used to secure model overfitting when constructing the model. DNN-based phantom shape scoring (DPS) models [multi-class classifier (MCC) and binary-class classifier (BCC)] were defined and the training set was used for MCC and BCC learning, and the validation set was used for those classifiers’ tuning. The relative loss and accuracy curves of training and validation according to all phantom shapes (fiber, speck, and mass) and DPS models (BCC, MCC) have been provided (supplementary Figure S1). The MCC has been identified as a three-class model in which each shape is directly assigned a score of 0, 0.5, or 1. When prediction is performed, the probability value for each class is finally obtained through the Softmax layer of the model, and the class having the highest probability is determined as the prediction score. For this reason, there is no threshold when performing prediction. The BCC has been identified as an additional model to more optimally match the 0.5 score class. BCC recognizes the 0.5 and 1 scores together as a class and further implements a binary classification model between this and the 0 score class. A probability value for a class with a score of 0.5 or 1 is obtained through the Sigmoid layer of the model. A probability value of 0.5 is considered the threshold corresponding to this class. A detailed network architecture and the whole training process has been provided (Fig. 4).

#### Statistical analysis of the models

The performance of the DPS models was evaluated using the holdout test set, which was the part of the development data set not used for training and validation. Additional performance was verified for the robustness of the model using an external validation set collected between May 1, 2018, and Mar 25, 2019, at two of our university-affiliated hospitals. We collected 70 phantom images (43 images for 10 months from hospital 1, 27 images for 7 months from hospital 2 with the identical mammography unit and phantom model), which were obtained for routine weekly phantom quality control. Nine phantom images, with acrylic discs obscuring details in the phantom, were excluded. Finally, the remaining 61 phantom images were scored in the same way as the development data set (Table 3).

**Table 3:** External validation set data status and score.

Score	61 Phantom images		
	No		
	Fibers	Specks	Masses
	366	305	305
0	156 (43%)	104 (34%)	117 (38%)
0.5	30 (8%)	9 (3%)	80 (27%)
1	180 (49%)	192 (63%)	108 (35%)

Accuracy was obtained from all combinations of scores and shapes for intuitive evaluation in each instance. The F1-score was calculated to evaluate and compare MCC and BCC performances. Area under receiver operating characteristic curve (AUC) analyses were additionally performed in order to evaluate BCC performances. The F1-score and AUC value were obtained through the classification_report API of the Python package scikit-learn v0.19.2. A 95% confidence interval (CI) was calculated by exact (Clopper-Pearson) confidence limits method using SAS v9.3 (SAS Institute, Cary, NC).

### Phantom image filtering using the models

#### Phantom image filtering algorithm (PFA)

**Figure 5:**
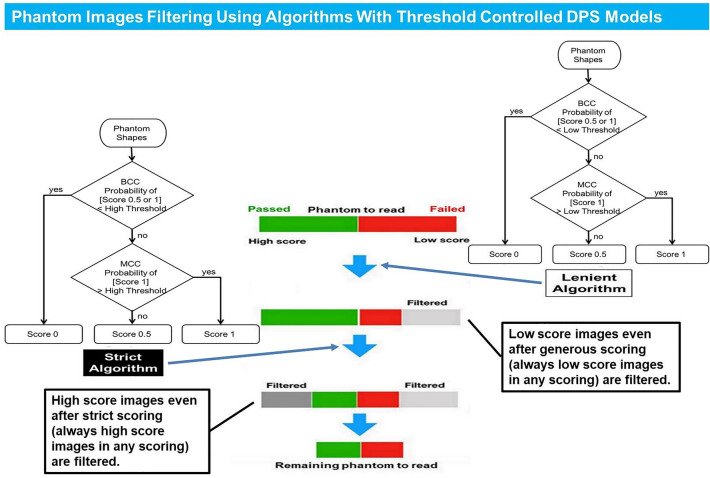
The phantom image filtering using the deep neural network based phantom shape scoring (DPS) models. The phantom images are filtered using two types of algorithms based on DPS models. The lenient algorithm filters phantoms that are to be determined as failed. The strict algorithm filters phantoms that are to be determined as passed.

The filtering algorithms were structured with two steps. When scoring a phantom shape, we selected a score of 0 or a score of (0.5 or 1) based on the specific threshold using a BCC probability of score (0.5 or 1). Second, in case the phantom shape was scored as (0.5 or 1), we selected a score of 0.5 or a score of 1 based on the specific threshold using an MCC probability of score 1. This was the clearest and simplest of the combinations that could be created using both BCC and MCC. We designed two types of algorithms based on this step. The lenient algorithm (LA) filters out phantom images expected to be a failure even with generous scoring. The strict algorithm (SA) filters out phantom images expected to pass even with strict scoring. We anticipated that these algorithms could safely reduce the need for assessment by a human observer by filtering out images that show the same result in any reading (Fig. 5).

#### Threshold for PFA and its application

**Figure 6:**
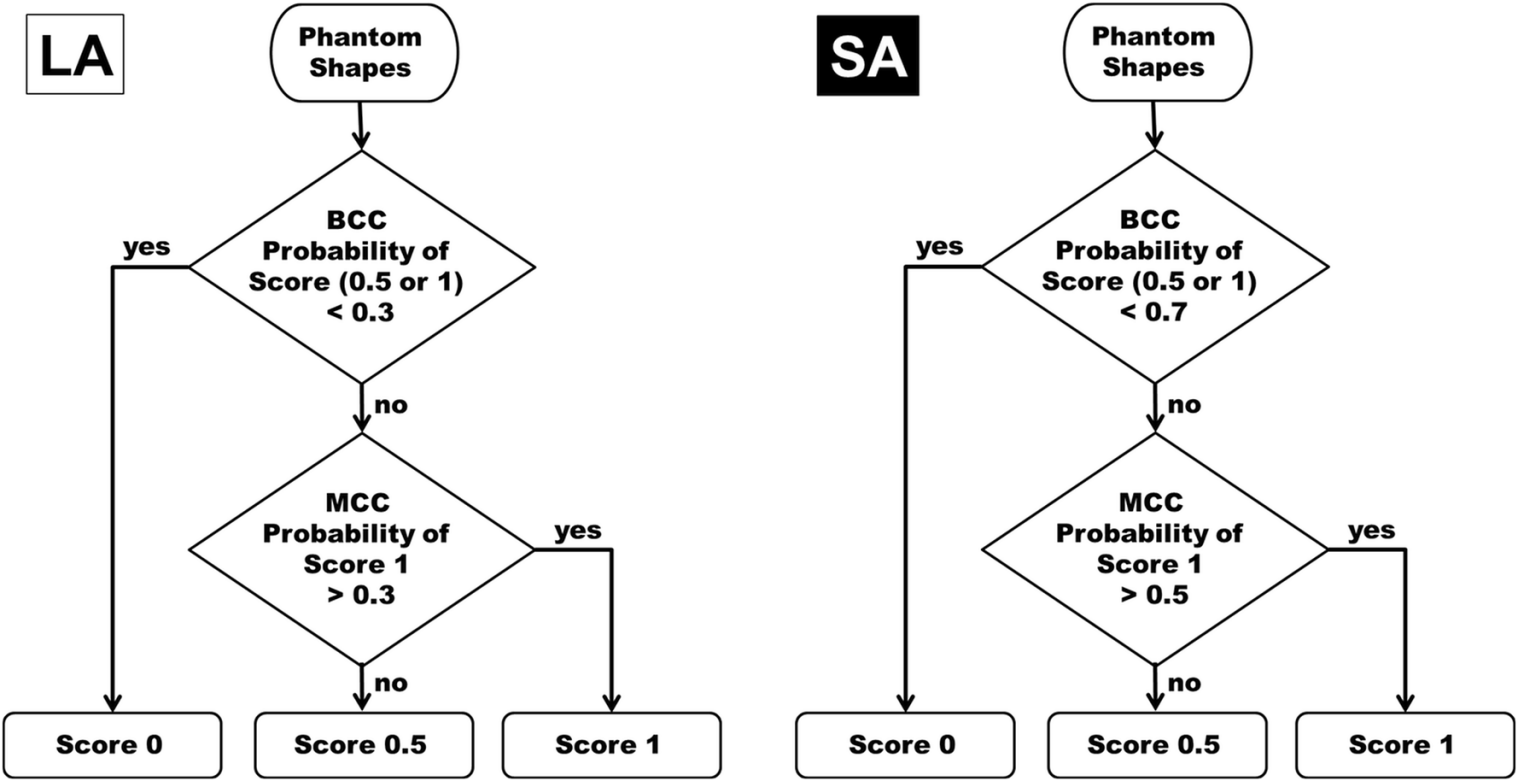
The final lenient algorithm (LA) and strict algorithm (SA) based on deep neural network based phantom shape scoring (DPS) models. Specific thresholds for LA and SA are selected after trying various combinations of threshold values.

In the case of the LA, the threshold was used in the expected lower range of 0.3–0.4 (BCC)/0.3–0.5 (MCC) for generous scoring. In the case of the SA, the threshold was implemented in the expected higher range of 0.6–0.7 (BCC)/0.4–0.6 (MCC) for strict scoring. After performing PFA with various combinations of threshold values with the 543 phantom images, the number of filtered images and the predictive value (hit/prediction ratio) were taken into consideration when selecting. Passing or failure of the phantom images was interpreted according to the ACR phantom scoring method^8^. If there is no longer a need for a human observer to assess a filtered image, accurate filtering should be considered more valuable than the number of filtered images. In order to meet this filtering purpose, we have chosen a threshold with excellent predictive value [LA 0.3 (BCC)/0.3(MCC), SA 0.7/0.5], even though the number of filtered images was reduced. Finally, while 277 out of 543 images were filtered with the selected threshold, 250 out of 277 images (90%) were ground-truth pass or fail results (Supplementary Table S5). Selected final algorithms are provided in Fig. 6. We applied the 61 phantom images for external validation to the final PFA in order to calculate how much filtering is actually possible.

## Results

The performances of the DPS model in our holdout set had an F1-score of 0.77 (95% CI 0.75, 0.80) for MCC and an F1-score of 0.95 (95% CI 0.94, 0.96) for BCC and an AUC of 0.98 (95% CI 0.97, 0.99) for BCC. For external validation, the DPS model showed slightly lower performance than the holdout set, i.e., F1-score of 0.69 (95% CI 0.65, 0.72) for MCC and F1-score of 0.93 (95% CI 0.92, 0.95) for BCC and AUC of 0.97 (95% CI 0.96, 0.98) for BCC (Table 4).

**Table 4:** Performance of the DPS models in the holdout and external validation set.

Performance	Holdout								External validation							
	MCC				BCC				MCC				BCC			
	Score	Fibers	Specks	Masses	Score	Fibers	Specks	Masses	Score	Fibers	Specks	Masses	Score	Fibers	Specks	Masses
Accuracy (%) (95% CI)	0	90.91 (85.66, 94.71)	88.1 (81.13, 93.18)	89.22 (83.5, 93.49)	0	88.64 (83, 92.92)	84.13 (76.56, 90.03)	88.62 (82.8, 93.01)	0	96.15 (91.82, 98.58)	94.23 (87.87, 97.85)	100 (96.9, 100)	0	85.9 (79.43, 90.95)	86.54 (78.45, 92.44)	99.15 (95.33, 99.98)
	0.5	30 (17.86, 44.61)	0 (0, 14.82)	61.69 (53.52, 69.4)	0.5 or 1	96.43 (94.34, 97.91)	98.08 (96.25, 99.17)	94.15 (91.28, 96.3)	0.5	0 (0, 11.57)	0 (0, 33.63)	23.75 (14.95, 34.58)	0.5 or 1	91.9 (87.36, 95.21)	99.5 (97.26, 99.99)	85.64 (79.8, 90.32)
	1	94.37 (91.73, 96.36)	99.24 (97.79, 99.84)	80.18 (74.32, 85.21)					1	95 (90.72, 97.69)	100 (98.1, 100)	86.11 (78.13, 92.01)				
Sensitivity, Recall (95% CI)			0.77 (0.74, 0.79)				0.93 (0.90, 0.95)				0.69 (0.66, 0.71)				0.90 (0.87, 0.92)	
PPV, Precision (95% CI)			0.79 (0.76, 0.81)				0.98 (0.96, 0.98)				0.85 (0.79, 0.90)				0.97 (0.95, 0.98)	
AUC (95% CI)			N/A				0.98 (0.97, 0.99)				N/A				0.97 (0.96, 0.98)	
F1-score (95% CI)			0.77 (0.75, 0.80)				0.95 (0.94, 0.96)				0.69 (0.65, 0.72)				0.93 (0.92, 0.95)	

DPS, deep neural network based phantom shape scoring; MCC, multi-class classifier; BCC, binary-class classifier; PPV, positive predictive value, N/A, not applicable; AUC, area under receiver operating characteristic curve.

Of the total 61 phantom images, 32 of the 38 failed images (84%) were filtered out using LA and 10 of the 23 passed images (43%) were filtered out by the SA. At the end, 42 of the 61 phantom images (69%) were filtered out by PFA. All of the phantom images predicted by failure through the LA produced a result of ground-truth failure, and all of the phantom images predicted by the pass through the SA showed ground-truth pass results (Table 5).

**Table 5:** Results after application of PFA in the external validation set.

PFA	61 Phantom images	
	Passed 23	Failed 38
Lenient algorithm		Failure hit/ Failure predict 32/32 (100%)
Strict algorithm	Pass hit/Pass predict 10/10 (100%)	
Total images filtered	10/23 (43%)	32/38 (84%)
	42/61 (69%)	

PFA, phantom image filtering algorithm.

## Discussion

### Machine learning and mammography phantom image

A considerable number of studies that have applied machine learning algorithms to full-field digital mammography are available. It can be broadly classified into computer-aided detection (CAD) systems, mammographic density assessment, and screening mammography triaging. In previous applications, large-scale data that can include the full range of findings (including various sizes, locations, and imaging findings) that can be seen in mammography is required to secure a high level of accuracy. In addition, these data should have included a wide variety of equipment, hospitals, age groups, and races. For the CAD system study, a “reader study” that proves the improvement of the radiologist's reading accuracy using the CAD system (improving breast cancer detection rate and reducing the recall rate in screening tests) is required in addition to evaluating the algorithm performance. Thus, performance evaluation in the actual breast cancer screening cohort must be made. For prospective cohort studies, it takes 1–2 years to recruit study subjects and a follow-up period of 1–2 years to evaluate interval cancer. Thus, it takes 3–4 years to obtain actual results. The application of machine learning algorithms to phantom images has several advantages. It is possible to create large-scale data by using a standardized phantom without any ethical problems. The size and position of the shape in the image are determined, and the image findings are relatively simple compared with the actual mammography. Furthermore, artificially matching various equipment (mammography unit/phantom) is possible. Age and race are irrelevant in all cases. Evaluating the reduction of manpower and time in actual clinical circumstances is necessary in addition to evaluating the algorithm performance, however, it will be very easy compared to a mammography study. Due to these advantages, if more machine learning approaches on phantom images can be made, it will be a field of machine learning research that is expected to have very satisfying results.

### Reliability of deep learning model and filtering algorithm

Several computerized assessment methods were published for the mammographic phantom image analysis^1–5^. We scored a phantom using a DNN, after learning the results of human observers as ground-truth. Considering that machine learning is a specialized sub-field of artificial intelligence, capable of learning and improving by studying high volumes of data for improved results, our attempt seems to be another suitable choice. The studies of Oh et al.^6^ and Ho et al.^7^ were also conducted based on this. A comparison with our study is provided (Table 1).

In our DPS model, proper training for a shape with a score of 0.5 was not performed due to relatively small number of samples with a score of 0.5. Consequently, MCC does not show enough performance (F1-score of 0.69, 95% CI 0.65, 0.72) in our external validation. We supplemented this weakness by creating a BCC (F1-score of 0.93, 95% CI 0.92, 0.95, AUC of 0.97, 95% CI 0.96, 0.98). If a sufficient number of a shape showing a score of 0.5 could be obtained, a high performance DPS model consisting only of an MCC would have been possible. The highest possible value of an F1-score is 1, indicating perfect precision and recall, and the AUC result is considered excellent for AUC values between 0.9 and 1. The performance of our BCC was very good, both in the holdout and the external validation set. Although binary classification, this high BCC performance means that only hundreds of phantom images (543 images) can make a high-performance DPS model. This also shows that DNN is highy promising as a computerized assessment method of the phantom shape. Although the F1-score of MCC was not as high as that of BCC, since our end goal was to filter the phantom images based on both BCC and MCC, the score was acceptable.

A PFA was developed using both BCC and MCC. Even if we could have high-enough performance, it is different from a perfect (meaning always correct) performance. There will be possible discordant results between the DPS models and ground-truth. Therefore, if pass/fail filtering is performed only by relying on high-performance DPS models, images of pass/fail that radiologists cannot agree on may be filtered. If there are many such results, a DNN-based scoring becomes unreliable. This is the reason we introduced PFA instead of simple DPS-based elimination. When we applied our PFA to our set of external validation images, the PFA was able to accurately exclude 69% of the phantom images by correctly identifying them as being either very likely to pass or very likely to fail a direct visual scoring by a radiologist. After filtering by the PFA, only less than one-third (31%) of the phantom images remained for direct visual scoring by radiologists. For all of the images that were filtered out by the PFA, there was 100% agreement between the algorithm-based pass/fail designation and the actual radiologist pass/fail interpretation. This result demonstrated that the PFA of our method is highly reliable. Even after multi-vendor/multi-reader study could be performed, DPS models with perfect performance would be impossible, so filtering through threshold-controlled algorithms as ours is more realistic.

In our study, ground-truth score of a phantom shape for machine learning was determined through consensus by two radiologists. Finding an accurate score as a ground-truth is not a problem that can be solved by many readers or majority decision; an accurate score may not be given by all of the readers. Therefore, judging the consensus scoring of two radiologists with their years of phantom scoring experience (17 and 9 years, respectively) is most realistic under our given conditions.

### Limitations and future considerations

Our study has several limitations. First, images were obtained from a single vendor’s mammography unit and phantom model. Hence, future studies should assess the generalizability of our results using multiple vendors’ mammography units and phantom models. Second, the sample size selected for the design of the study and the external validation was “convenience sampling”. However, we tried to obtain as much sample data as possible within a given set of circumstances. Future studies are expected to better solve the overfitting problem that can occur with small amounts of data by applying a learning algorithm such as stochastic weight averaging. In future studies, careful consideration should be given to the determination of unbiased training and validation set size. We could perhaps examine whether learning curves (accuracy versus size of training data) would suggest whether more data would help. Third, further studies require steps that measure the optical density and detect the artifact prior to scoring the phantom shape. This will help improve the ability to exclude more images.

In spite of the limitations, our trial shows that it is possible to exclude a sufficient number of images through the threshold-controlled algorithm based on a DNN. For people who are not fluent in phantom reading, this can be a reference standard to maintain the objectivity of the results, and a double reading effect can also be anticipated. When a PFA is developed with an easy-to-use software that works with a picture archiving communication system, our algorithm may reduce the workload of the radiology department and would ultimately be beneficial for providing more quality hospital services. Our final goal is to develop phantom filtering software and to confirm the effect (reduction of time or manpower) that appears when it is used in actual clinical situation. The development of an algorithm that reduces staff workload by filtering a passed or failed phantom image based on a DNN has not been attempted until now.

The VGG16 is a model of image data for image recognition and is well known for its good image feature extractor. However, a study of other architectures and training procedures plus ideally “ablation study” indicating what choices impact the result the most will likely be useful as we move beyond a single hospital study into a multi-center trial. We used the Softmax / Sigmoid output of the model as a measure of certainty. However, this may not be an ideal way to filter the data, as it does not properly capture model uncertainty. In future studies, adding more robust measures, such as dropout-based uncertainty measures, will be helpful.

According to the method we proposed, each shape must be cropped out of a phantom image, and the cropped shape must be further preprocessed before passing through PFA. In its current state, it takes much more time than does actual visual scoring. In the future, even if we make software that can perform all tasks automatically in a given phantom image, it is not easy to exceed the speed of visual scoring. However, our goal is not to reduce the scoring time per image. Our goal is to filter out definite passed or failed phantom images ahead of visual scoring, thereby ultimately reducing human effort. The automated software should operate without time constraints, so radiologists will have a reduced number of images to score. This will be more useful in accrediting agencies that conduct a large number of phantom scoring.

## Conclusions

We developed a reliable DNN-based algorithm to reduce the workload in mammographic phantom interpretation and demonstrated its feasibility. Further studies are necessary to determine the efficacy and feasibility of applying the automated DNN-based algorithm to the mammographic phantom interpretation in clinical practice. In the future, a specific DNN model using VGG16 as the foundational base could be developed.

## Supplementary Information

Supplementary Tables.

Supplementary Figures.

## Data Availability

The authors declare that the main data supporting the findings of this study are available within the article and its Supplementary files. Extra data are available from the corresponding author on reasonable request.
