# Supplementary material for: Devising a deep neural network based mammography phantom image filtering algorithm using images obtained under mAs and kVp control
Source: Sci Rep. 2023 Mar 2;13:3545. doi: 10.1038/s41598-023-30780-z (PMC9981722; doi:10.1038/s41598-023-30780-z)
Supplement: Supplementary file 1 — Supplementary Tables. [file 41598_2023_30780_MOESM1_ESM.docx]

**Supplementary Table S1.** Distribution of 640 phantom images created at different mAs / kVp.

|  | **kVp** | | | | | | | | | | | |
| --- | --- | --- | --- | --- | --- | --- | --- | --- | --- | --- | --- | --- |
| **mAs** | **22** | **23** | **24** | **25** | **26** | **27** | **28** | **29** | **30** | 31 | 32 |  |
| 60- | 1 | 3 | 4 | 4 | 4 | 4 | 4 | 4 | 4 | 4 | 4 |  |
| 70- | 2 | 3 | 4 | 5 | 5 | 4 | 4 | 4 | 5 | 4 | 5 |  |
| 80- | 3 | 3 | 5 | 5 | 4 | 5 | 5 | 5 | 4 | 5 | 4 |  |
| 90- | 3 | 3 | 3 | 3 | 3 | 3 | 3 | 3 | 3 | 3 | 3 |  |
| 100- | 3 | 3 | 3 | 3 | 3 | 3 | 3 | 3 | 3 | 3 | 3 |  |
| 110- | 3 | 3 | 4 | 4 | 4 | 3 | 4 | 4 | 4 | 3 | 4 |  |
| 120- | 3 | 4 | 4 | 4 | 4 | 4 | 4 | 4 | 4 | 4 | 4 |  |
| 130- | 3 | 4 | 3 | 3 | 4 | 4 | 3 | 4 | 3 | 4 | 4 |  |
| 140- | 3 | 3 | 3 | 3 | 3 | 4 | 3 | 3 | 3 | 3 | 3 |  |
| 150- | 3 | 4 | 4 | 4 | 3 | 3 | 4 | 3 | 4 | 3 | 3 |  |
| 160- | 4 | 3 | 4 | 4 | 3 | 3 | 4 | 3 | 4 | 4 | 3 |  |
| 170- | 4 | 3 | 2 | 2 | 3 | 3 | 2 | 3 | 2 | 3 | 3 |  |
| 180- | 4 | 5 | 5 | 5 | 5 | 5 | 5 | 5 | 5 | 5 | 4 |  |
| 190- | 3 | 4 | 4 | 4 | 5 | 4 | 4 | 5 | 4 | 5 | 4 |  |
| 200- | 3 | 5 | 5 | 4 | 4 | 5 | 5 | 4 | 5 | 4 | 3 |  |
| 210- | 3 | 3 | 3 | 4 | 3 | 3 | 3 | 3 | 3 | 3 | 1 |  |
| mAs, milliampere-seconds; kVp, kilovoltage peak. | | | | | | | | | | | |  |

**Supplementary Table S2.** A phantom image scoring criteria.

| **Fibers** |
| --- |
| A minimum of the four largest fibers in their entirety (four of six) should be seen. The entire fiber must be seen to give it a score of 1; if a fiber cannot be completely seen, that fiber may be scored 0.5. Start by counting the largest fiber. Continue scoring the individual fibers, adding the results together and moving from larger toward smaller as long as a full score (whole number) is achieved. Once a partial score (0.5) is given, a smaller fiber may not be counted. |
| **Speck groups** |
| A minimum of the three largest speck groups (three of five) should be seen. Four to six specks in a group must be seen to give it a score of 1; if only two or three specks out of six in a group are seen, that group may be scored 0.5. Start by counting the number of specks in the largest group. Continue scoring the speck groups, adding the results together and moving from larger toward smaller as long as a full score (whole number) is achieved. Once a partial score (0.5) is given, a smaller speck group may not be counted. |
| **Masses** |
| A minimum of the three largest masses (three of five) should be seen. If both a density difference and the entire circumscribed edge can be seen, it will receive a score of 1; if only the density difference or part of the edge is seen, that mass may be scored 0.5. Start by evaluating the largest mass. Continue scoring the masses, adding the results together and moving from larger toward smaller as long as a full score (whole number) is achieved. Once a partial score is given (0.5), a smaller mass may not be counted. |

**Supplementary Table S3.** Performance comparison in hold-out set between several models.

|  |  | VGG16 | | ResNet50 | | MobileNet | |
| --- | --- | --- | --- | --- | --- | --- | --- |
|  |  | Accuracy (%) | AUC (0~1) | Accuracy (%) | AUC (0~1) | Accuracy (%) | AUC (0~1) |
| MCC | Fibers | **87.58** | - | 83.59 | - | 82.06 | - |
|  | Specks | **92.08** | - | 91.90 | - | 81.40 | - |
|  | Masses | **80.48** | - | 77.35 | - | 74.77 | - |
| BCC | Fibers | **94.94** | **0.987** | 86.50 | 0.946 | 90.64 | 0.964 |
|  | Specks | **95.03** | **0.990** | 94.66 | 0.983 | 84.16 | 0.913 |
|  | Masses | **93.74** | **0.987** | 91.71 | 0.975 | 88.40 | 0.951 |

**Supplementary Table S4.**Performance comparison(VGG16) in hold-out set, pre-training vs. training from scratch.

|  |  | Pre -Training | | Training from scratch | |
| --- | --- | --- | --- | --- | --- |
|  |  | Accuracy (%) | AUC (0~1) | Accuracy (%) | AUC (0~1) |
| MCC | Fibers | 87.58 | - | **87.88** | - |
|  | Specks | **92.08** | - | 91.34 | - |
|  | Masses | **80.48** | - | 80.11 | - |
| BCC | Fibers | **94.94** | **0.987** | 94.63 | 0.987 |
|  | Specks | **95.03** | **0.990** | 94.84 | 0.988 |
|  | Masses | **93.74** | **0.987** | 91.53 | 0.979 |

The use of a pre-training network is slightly better than training from scratch (reset weight).


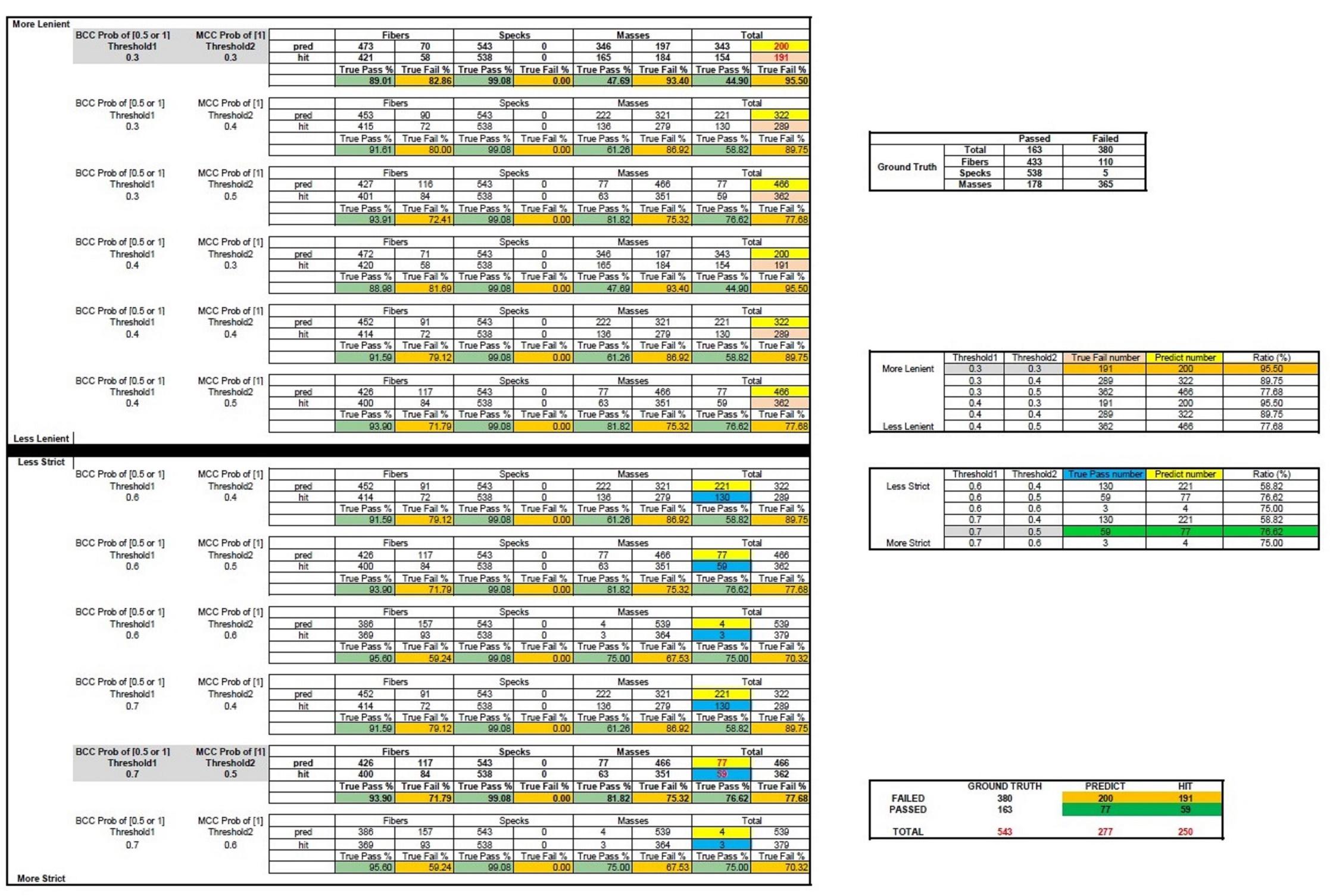


**Supplementary Table S5.** The threshold selection for phantom image filtering algorithm (PFA). PFA is performed with various combination of threshold values at the generated 543 phantom images.
