# Supplementary material for: Devising a deep neural network based mammography phantom image filtering algorithm using images obtained under mAs and kVp control
Source: Sci Rep. 2023 Mar 2;13:3545. doi: 10.1038/s41598-023-30780-z (PMC9981722; doi:10.1038/s41598-023-30780-z)

Supplementary Figure S1. Loss and Accuracy Curves (Training and Validation) in different shapes and DPS models.

BCC_Fibers

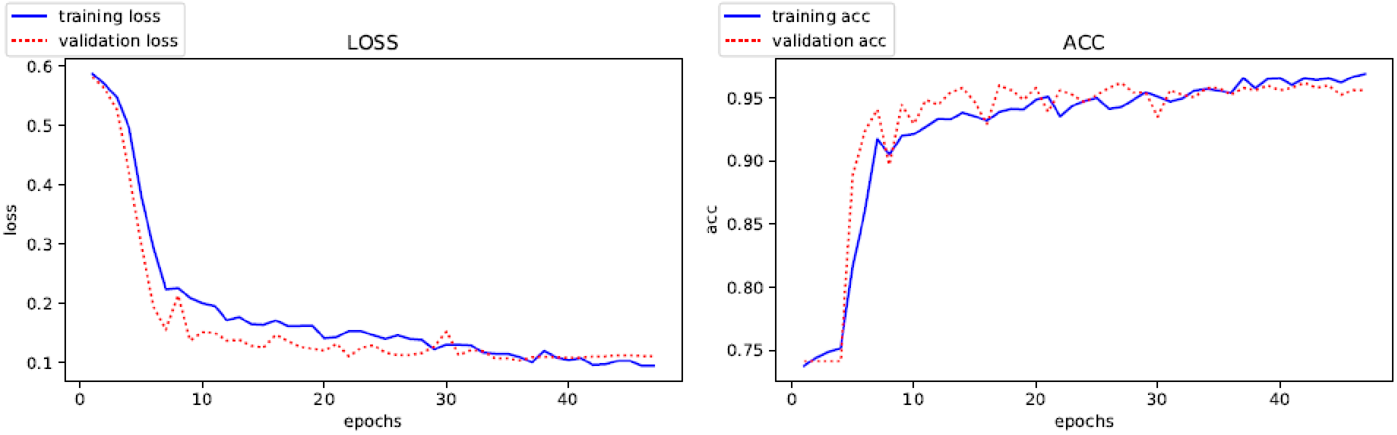

BCC_Specks

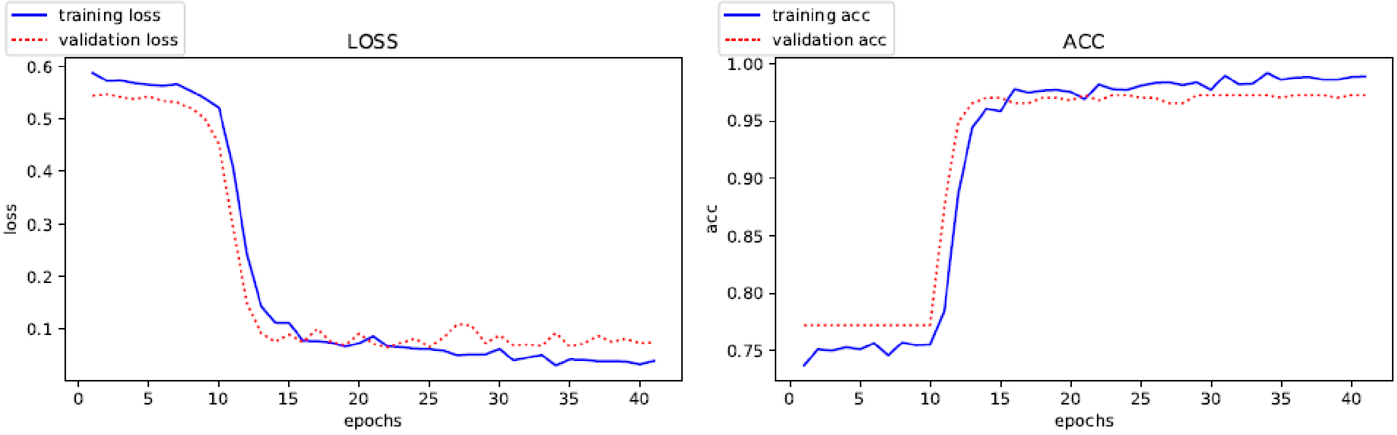

BCC_Masses

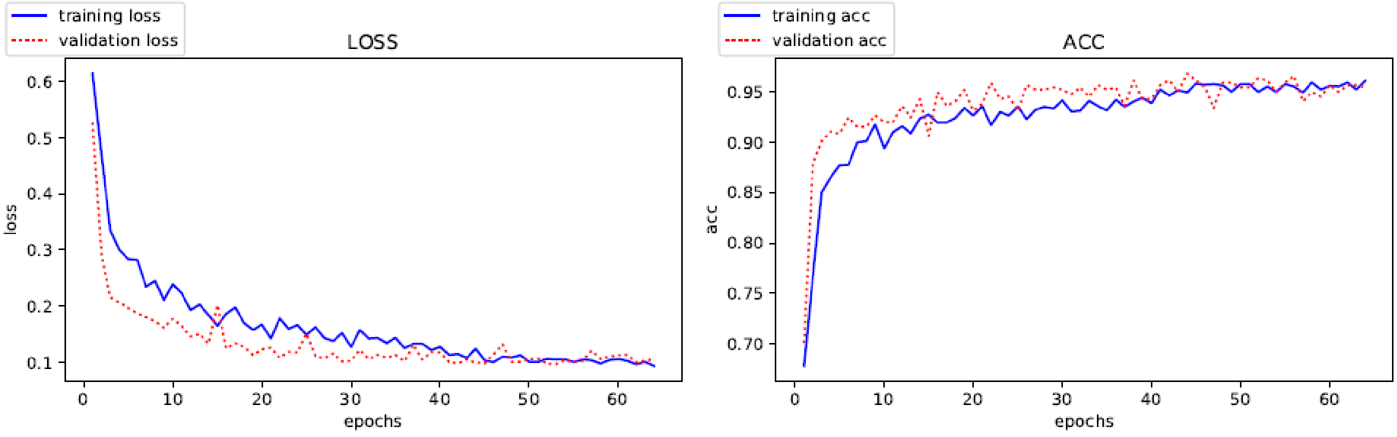

MCC_Fibers

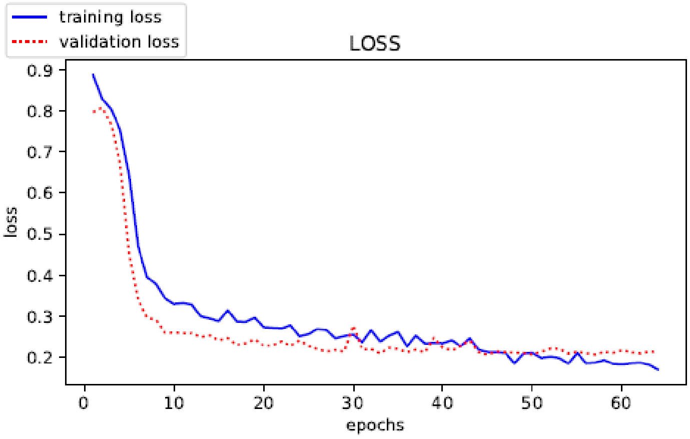

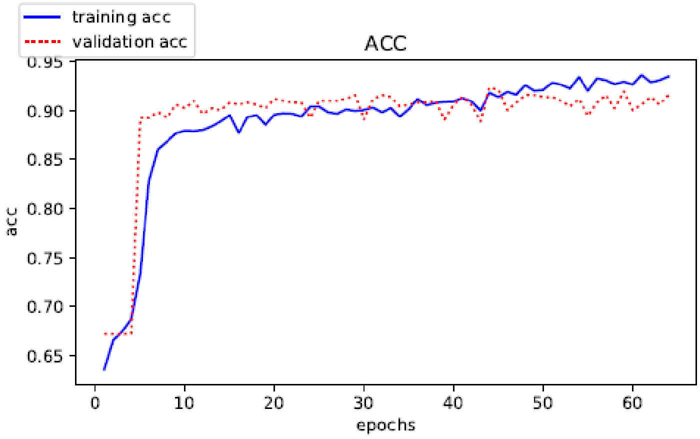

MCC_Specks

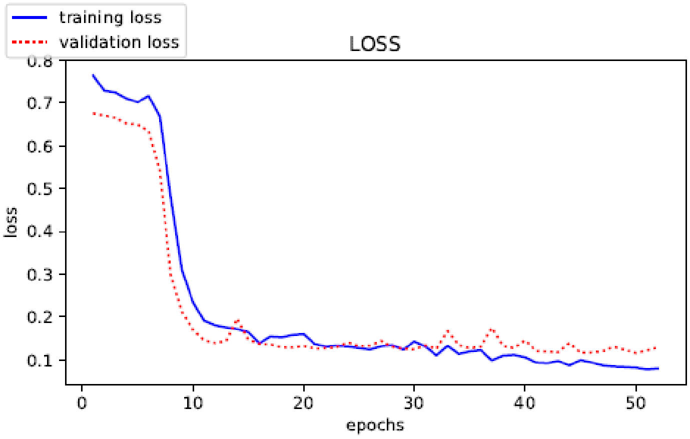

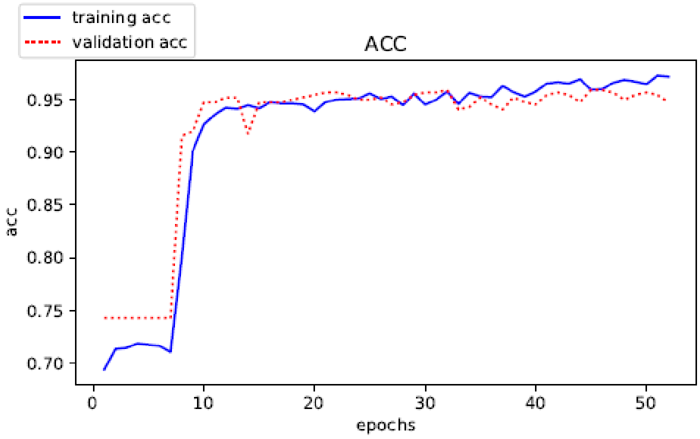

MCC_Masses

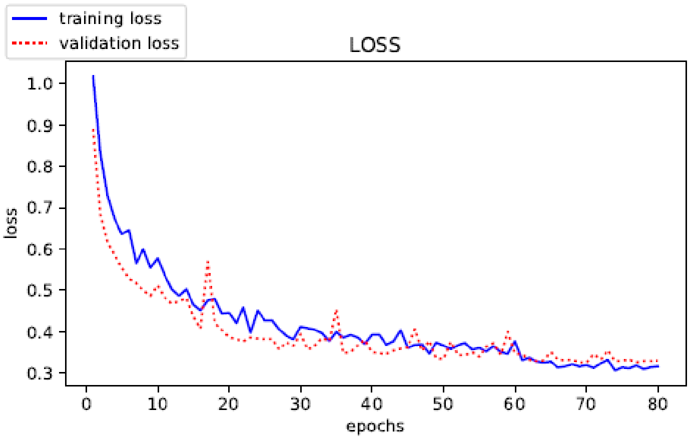

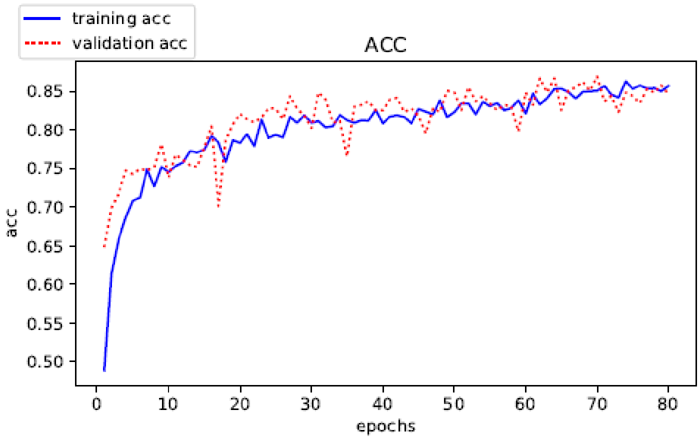

Supplement: Supplementary file 2 — Supplementary Figures. [file 41598_2023_30780_MOESM2_ESM.pdf]
